# Analysis of optimal volume fraction percentage and influencing factors of bone cement distribution in vertebroplasty using digital techniques

**DOI:** 10.1186/s13018-023-03719-3

**Published:** 2023-03-23

**Authors:** Chengqiang Zhou, Yifeng Liao, Han Chen, Yunqing Wang

**Affiliations:** 1 Department of Orthopedics, The Second Affiliated Hospital of Xuzhou Medical University, 32 Meijian Road, Xuzhou, 221000 Jiangsu China; 2 Graduate School of Xuzhou Medical University, Xuzhou, 221000 Jiangsu China

**Keywords:** Percutaneous vertebroplasty, Osteoporosis, Spinal fractures, Bone cement distribution, Cement dispersion volume, Aged

## Abstract

**Purpose:**

To explore the optimal volume fraction percentage (VF%) and influencing factors of bone cement distribution in percutaneous vertebroplasty (PVP) for osteoporotic vertebral compression fractures (OVCF) using digital techniques.

**Patients and methods:**

From January 2019 to February 2021, 150 patients with 0VCF who underwent PVP surgery in our hospital were analyzed. Based on postoperative X-ray and CT, the spatial distribution score of the intravertebral cement was calculated and the patients were divided into two groups: 0–7 were divided into group A; 8–10 were divided into group B. The general data of the two groups of patients were compared, and Mimics three-dimensional reconstruction images were used to measure the cement dispersion volume (CDV), vertebral body volume (VBV), and VF%. Factors affecting bone cement distribution were included in a multifactorial logistic regression analysis to construct a receiver operating characteristic (ROC) curve, calculate a cut-off value for the extensive distribution of bone cement, and analyze the correlation between bone cement distribution scores and VF%.

**Results:**

There were 60 patients in group A and 90 patients in group B. Univariate analysis showed that bone mineral density (BMD), cement leakage, CDV, and VF% were significantly lower in group A than in group B (*p* < 0.05). Multivariate logistic regression analysis showed that BMD and VF% were independent influencing factors on bone cement distribution. The area under the curve (AUC) of VF% was 84.7%, and the cut-off value for extensive distribution of bone cement was 28.58%, which corresponded to a sensitivity and specificity of 72.2% and 91.7%, respectively. There was a strong correlation between the cement distribution score and VF% (*r* = 0.895, *p* < 0.001).

**Conclusion:**

BMD and VF% were important independent influencing factors of bone cement distribution. Extensive bone cement distribution can be achieved when the VF% reaches 28.58%.

## Introduction

Elderly osteoporotic populations are prone to osteoporotic vertebral compression fractures (OVCF) [1, 2]. The prevalence of OVCF has been reported to be as high as 20% in people over 50 years of age [3, 4], posing a significant medical burden to families and society. Percutaneous vertebroplasty (PVP) or percutaneous kyphoplasty (PKP) has become an important technical measure for the clinical treatment of OVCF [5, 6]. Bone cement is the main filling material in the vertebral body during PVP surgery. Its distribution in the vertebral body is closely related to the curative effect after surgery [7]. However, the diffuse distribution pattern of bone cement within the vertebral body is complex and diverse, and there is a lack of accepted criteria for judging the distribution. In addition, there are still few studies on the influencing factors of bone cement distribution.

Mimics 21.0 (Materiallise softwar, Belgium) software is an image processing tool that can convert two-dimensional image data into three-dimensional (3D) image data and is widely used in the field of digital medicine, allowing physicians to visualize the converted 3D model more intuitively and accurately. Therefore, in this context, we applied Mimics software to evaluate the degree of bone cement dispersion in order to more accurately evaluate the cement dispersion volume (CDV), vertebral body volume (VBV), and volume fraction percentage (VF%). This study retrospectively analyzed 150 patients with OVCF treated with PVP in our orthopedic department from January 2019 to February 2021 to investigate the optimal VF% and influencing factors of cement distribution during vertebroplasty and to study the correlation between cement distribution score and VF%.

## Materials and methods

### General data

Inclusion criteria: (1) single-segment OVCF patients with significant low back pain; (2) bone mineral density (BMD) T value ≤ -2.5; (3) PVP; (4) compression ratio of the injured vertebra ≤ 1/3; (5) MRI examination of the injured vertebra showed high signal in T2W1, and edema signal existed in fat suppression sequence imaging; (6) Complete clinical data, imaging data, and follow-up data were available. Exclusion criteria: (1) pathological fracture due to tumor or infection; (2) combined with serious cardiac, pulmonary, hepatic, and renal insufficiency; (3) unable to tolerate surgery prone; (4) with coagulation dysfunction; (5) cement leakage in the spinal canal or obvious leakage in the paravertebral and intervertebral spaces; (6) patients with less than one year of follow-up.

### Surgical method

The patient was positioned prone, with the chest and shoulders and pelvis elevated with padded pillows and the head and tail of the surgical bed moderately elevated so that the treatment site was in a posteriorly extended position with the abdomen suspended. The puncture site was determined by fluoroscopy with a G-arm x-ray machine. 1% lidocaine was used for local infiltration anesthesia after disinfection and spreading of the towel. The puncture needle was punctured into the anterior middle and lower 2/3 of the injured vertebrae via the pedicle, and the middle and later stages of polymethylmethacrylate bone cement were slowly injected in fractions under fluoroscopy. Postoperatively, the patient was lying flat for at least 2 h. Anti-osteoporotic drugs were routinely administered for treatment and functional exercise.

### Evaluation method

**Fig. 1:**
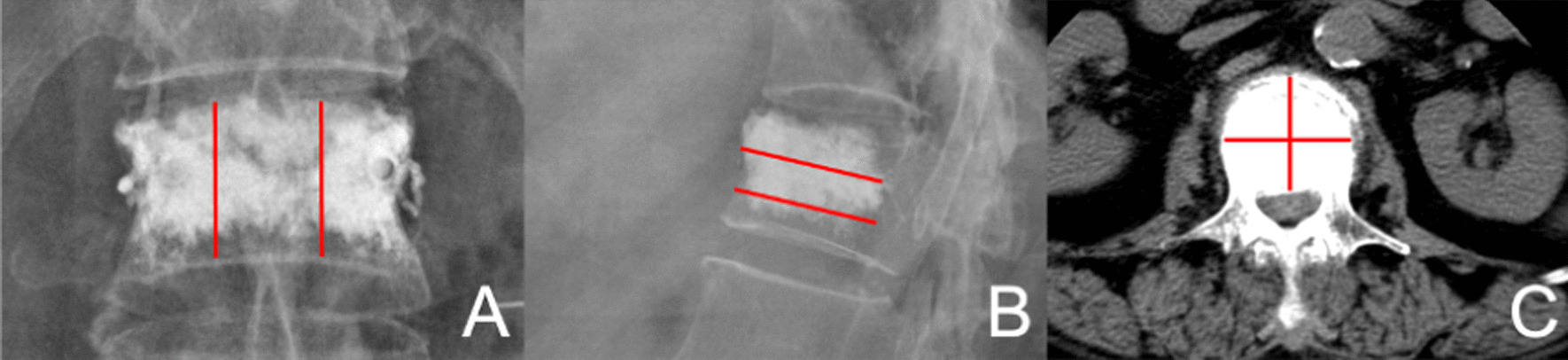
Imaging score of the bone cement distribution **A** Coronal plane, **B** Sagittal plane, **C** Horizontal plane

The distribution scores in the coronal and sagittal planes were calculated based on the cement distribution characteristics on the postoperative frontal and lateral x-ray images, and the distribution scores in the horizontal plane were calculated based on the cement distribution on the postoperative CT images at different levels. Coronal and sagittal scoring: On the frontal and lateral x-ray images of the vertebral body, two vertical and horizontal lines were drawn through the coronal and sagittal planes to divide the vertebral body into three equal parts: left, middle, and right; and upper, middle, and lower (Fig. 1A and B). Score 3: Bone cement was distributed in three areas and as close as possible to the left and right walls as well as the upper and lower endplates. Score 2: Bone cement was distributed in two of the areas. Score 1: Bone cement was distributed in one of the areas. Score 0: Bone cement was distributed in less than 50% of the areas in all three areas. Horizontal plane score: The vertebral body was divided into four areas by making horizontal and vertical lines through the center of the horizontal plane of the vertebral body CT, and the bone cement distribution score was observed and calculated (Fig. 1C). Score 4: Bone cement was distributed in four areas. Score 3: Bone cement was distributed in three of the areas. Score 2: Bone cement was distributed in two of the areas. Score 1: Bone cement was distributed in one of the areas. Score 0: Bone cement was distributed in less than 50% of the area in all four areas on the horizontal plane. The horizontal scores were counted in the plane with the highest score, and then added to the sagittal and coronal scores to obtain the bone cement spatial distribution score and divided into two groups: Group A, 0–7 scores, with limited distribution of cement in the vertebral body; Group B, 8–10 scores, with extensive distribution of cement in the vertebral body [8]. Sixty patients had a limited distribution in group A and ninety patients had an extensive distribution in group B.

### Calculation of the VF%

**Fig. 2:**
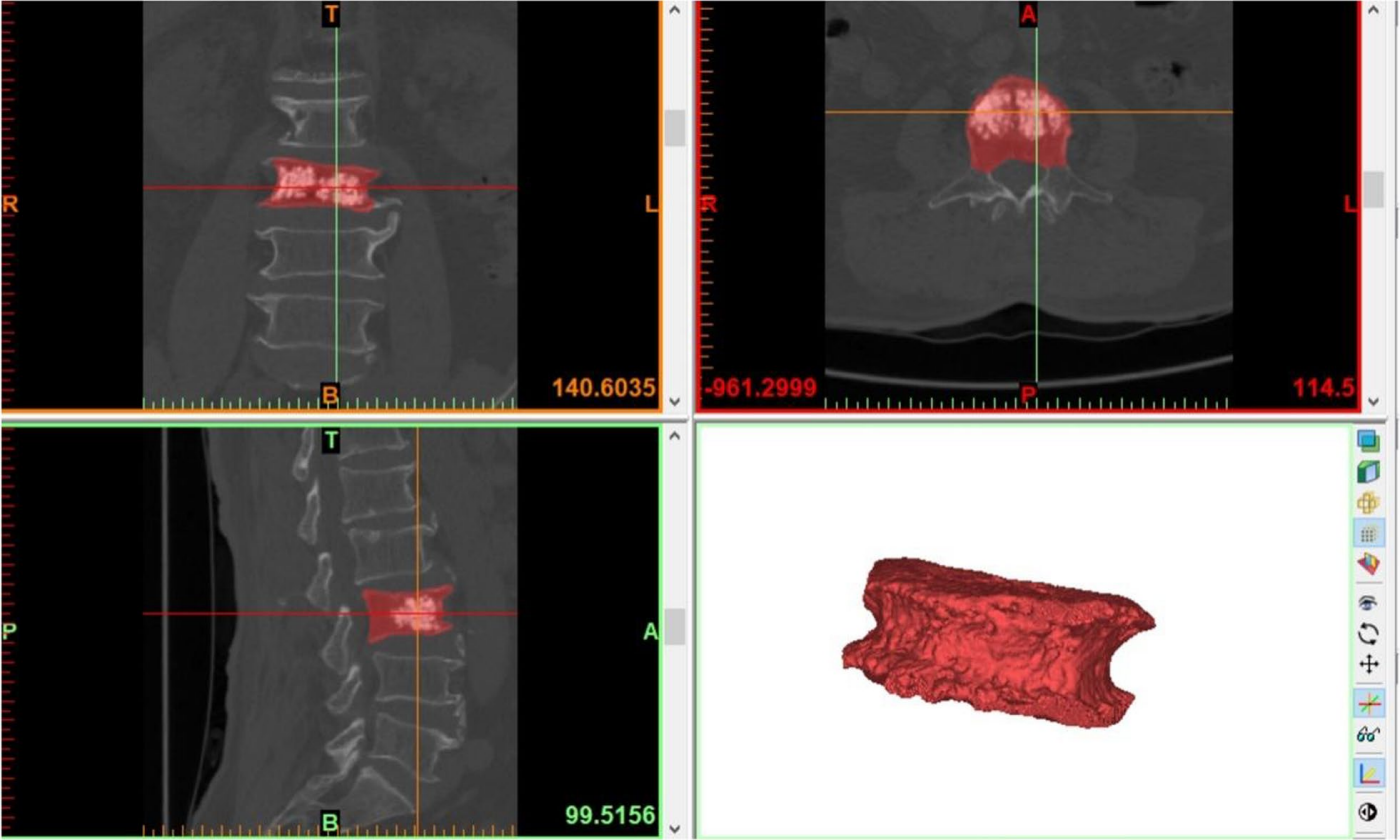
3D model of fractured vertebrae. (T, top; B, bottom; R, right; L, left; A, anterior; P, posterior)

**Fig. 3:**
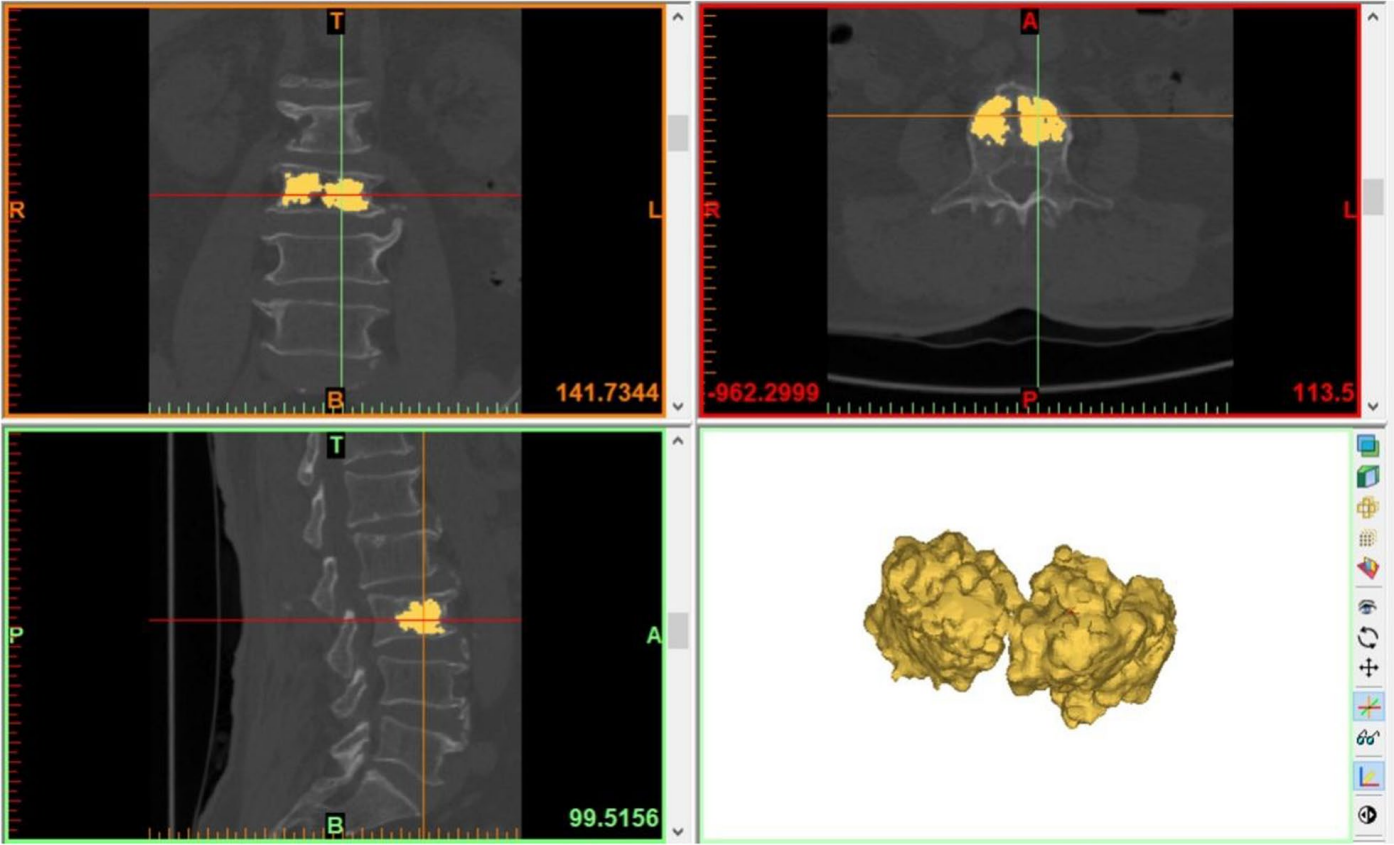
3D model of bone cement. (T, top; B, bottom; R, right; L, left; A, anterior; P, posterior)

① CT data collection: All patients underwent CT scans before surgery and 3 days after surgery, and the scanning conditions were met: 120 kV, 250 mA. Scan with the diseased vertebra as the center, and set the slice thickness to 0.625 mm. The CT examination data were then exported to DICOM file format.
② Mimics software calculation: The acquired DICOM data were imported into Mimics 21.0 software to create a Mimics project file. The type of dataset was 32-bit. The Slice Increment and Slice Thickness resolutions of the image were both 0.4 mm. The bone tissue was separated and extracted with the threshold segmentation tool, and the reference threshold range was the system default threshold for bone tissue definition (226–3071Hu). The software automatically generates contour lines of the bone tissue surface layer by layer, and then uses the 3D reconstruction function to obtain a 3D model of the spinal segment in this region. The Mask Edit tool was used to remove the bilateral transverse processes, pedicles, and laminae, and then the gaps were repaired with the void filling function to obtain a fractured vertebral body model (Fig. 2). The VBV was calculated to be 26.28 cm3. Then the threshold value was adjusted to the bone cement threshold (1000–3000Hu), and the bone cement was extracted by separating the bone tissue from the bone cement threshold, and the 3D model of bone cement was obtained by using the 3D reconstruction function. The Mask Edit tool was used to remove the portion of the bone cement model that leaked outside the vertebral body to obtain a bone cement model (Fig. 3). The CDV was calculated to be 7.63 cm3. Finally, the VF% was calculated as 29.03% based on the CDV/VBV calculation. The above data were processed and calculated separately by three spine surgeons skilled in the use of Mimics software, and finally the average of the three was taken.

### Observation indexes

Gender, age, BMI, BMD, fracture segment, cement leakage, and operation time were recorded and compared between the two groups, and CDV, VBV, and VF% were calculated.

### Statistical analysis

All the data from this study were analyzed using SPSS 26.0 statistical software (IBM, Armonk, NY, USA). The inter- and intra-observer reliability of the measurements were assessed by intraclass correlation coefficients (ICCs). Data were obtained postoperatively by 3 experienced surgeons. An ICC value > 0.75 mean high reliability. The measurement data conforming to normal distribution were expressed as _χ ± s using the t-test; the count data were expressed as rates (%) using the χ2 test. The relevant variables (*p* < 0.05) with potential influence on bone cement distribution were subjected to multivariate logistic regression analysis, and receiver operating characteristic (ROC) curves were constructed to calculate the cut-off value for the extensive distribution of bone cement. A Spearman correlation analysis was used to analyze the correlation between bone cement distribution scores and VF%, and correlation coefficients > 0.7 were deemed strong correlations. *p* < 0.05 was considered statistically significant.

## Results

### General information

All patients completed the surgery successfully, and 150 patients were followed up for more than one year without serious surgical complications. Bone cement distribution score statistics: two cases with 2 scores; five cases with 3 scores; seven cases with 4 scores; 10 cases with 5 scores; 15 cases with 6 scores; 21 cases with 7 scores; 37 cases with 8 scores; 32 cases with 9 scores; and 21 cases with 10 scores.

The ICCs were found to be greater than 0.90 in all three surgeons for both inter- and intra-observer reliability, which indicates high reliability.

### Univariate analysis of bone cement distribution

There were no significant differences in gender, age, BMI, fracture segment, operation time, and VBV between the two groups (*p* > 0.05); BMD, cement leakage, CDV, and VF% were significantly lower in group A than in group B (*p* < 0.05) (Table 1).

**Table 1:** Univariate analysis affecting the distribution of bone cement

Factors	Group A(60)	Group B(90)	t/x^2^	*p*
Gender			0.125	0.724
Male	21(35.0%)	29(32.2%)		
Female	39(65.0%)	61(67.8%)		
Age(years)	71.12 ± 7.14	72.69 ± 7.52	− 1.280	0.203
BMI (kg/m^2^)	23.13 ± 3.92	23.35 ± 3.59	− 0.360	0.720
BMD (T score)	− 3.23 ± 0.43	− 3.06 ± 0.53	− 2.204	0.029
Fracture segment			1.435	0.488
T1–T10	14(23.3%)	14(15.5%)		
T11–L2	37(61.7%)	61(67.8%)		
L3–L5	9(15.0%)	15(16.7%)		
Cement leakage			4.202	0.040
Leakage	12(20.0%)	32(35.6%)		
No leakage	48(80.0%)	58(64.4%)		
Operation duration (mins)	43.37 ± 4.05	44.33 ± 5.03	− 1.297	0.197
CDV (ml)	6.39 ± 1.90	8.13 ± 2.15	− 5.216	< 0.001
VBV (ml)	26.49 ± 5.70	26.15 ± 6.74	0.322	0.748
VF% (%)	24.46 ± 6.33	31.44 ± 5.22	− 7.366	< 0.001

BMI, body mass index; BMD, bone mineral density; CDV, cement dispersion volume; VBV, vertebral body volume; VF%, volume fraction percentage

### Multivariate analysis of bone cement distribution

Multivariate logistic regression analysis showed that BMD (OR = 2.649, 95% CI = 1.154 to 6.083, *p* = 0.022) and VF% (OR = 1.259, 95% CI = 1.135 to 1.396, *p* < 0.001) were independent risk factors affecting the bone cement distribution. In contrast, CDV and cement leakage are not influential factors for bone cement distribution (Table 2).

**Table 2:** Multivariate analysis affecting the distribution of bone cement

Factors	OR	95%CI		P
		Lower	Upper	
BMD	2.649	1.154	6.083	0.022
Cement leakage	2.361	0.932	5.978	0.070
CDV	1.173	0.940	1.463	0.158
VF%	1.259	1.135	1.396	< 0.001

BMD, bone mineral density; CDV, cement dispersion volume; VF%, volume fraction percentage; OR, odds ratio; CI, confidence interval

### ROC curve analysis of bone cement distribution

**Fig. 4:**
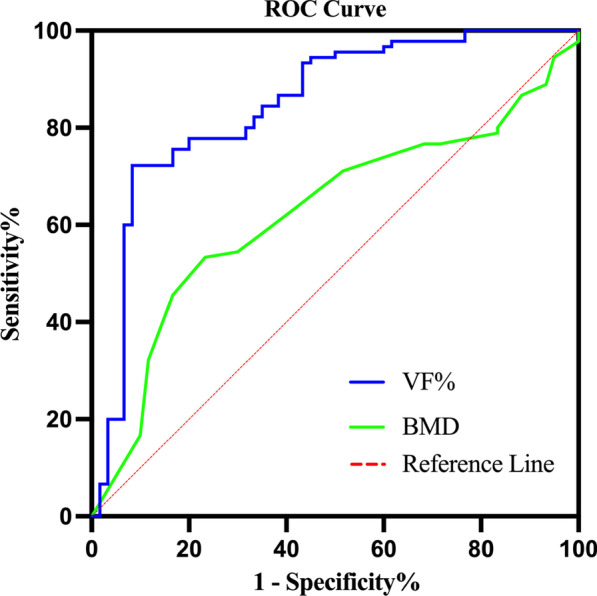
ROC curves of BMD and VF%. BMD, bone mineral density. VF%, volume fraction percentage

The ROC curves for BMD and VF% were plotted (Fig. 4). The area under the curve (AUC) of BMD was 0.625 (95% CI: 0.534–0.716, *p* = 0.010); the AUC of VF% was 0.847 (95% CI: 0.780–0.914, *p* < 0.001) (Table 3).

**Table 3:** AUC of BMD and VF%

Factors	AUC(Area)	P	95%CI	
			Lower	Upper
BMD	0.625	0.010	0.534	0.716
VF%	0.847	< 0.001	0.780	0.914

BMD, bone mineral density; VF%, volume fraction percentage; AUC, area under the curve; CI, confidence interval

### Cut-off values for the extensive distribution of bone cement

The cut-off value for the extensive distribution of bone cement was 28.58%, which corresponds to a sensitivity and specificity of 72.2% and 91.7%, respectively (Table 4).

**Table 4:** The best cut-off value of extensive distribution of bone cement

Factor	Cut-off value	Sensitivity	Specificity
VF%	28.58	0.722	0.917

VF%, volume fraction percentage

### Relationship between cement distribution score and VF%

**Fig. 5:**
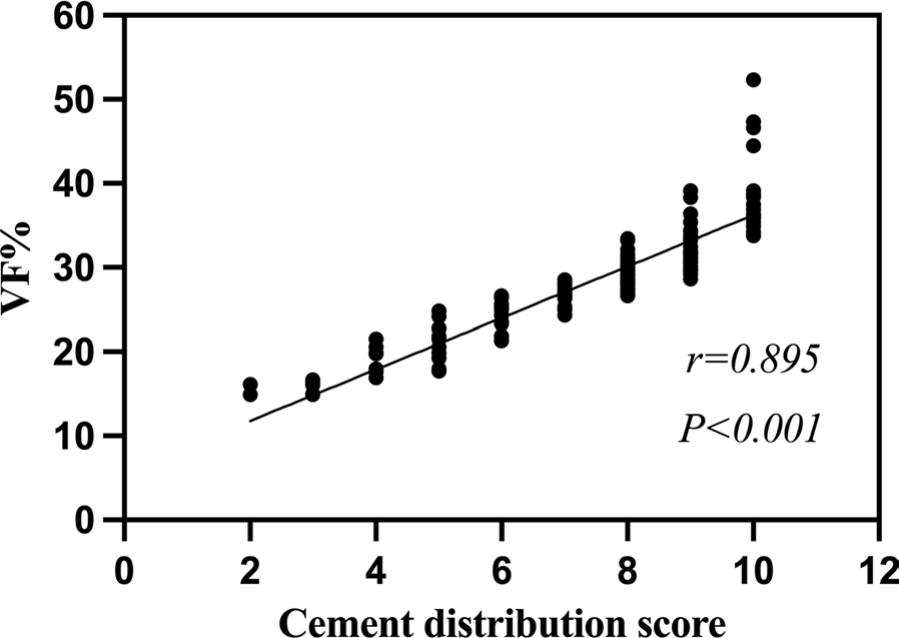
Scatter plot of correlation between cement distribution score and VF%. *Notes*: *r* > 0.7 indicates a strong correlation between the two. VF%, volume fraction percentage

The mean VF% was (28.65 ± 6.63) % in 150 patients. Correlation analysis showed a strong correlation between the cement distribution score and the VF% (*p* < 0.001, *r* = 0.895). The scatter plot is shown in Fig. 5.

## Discussion

OVCF is a common complication of osteoporosis, which can present with severe pain in the thoracic and lumbar back, loss of vertebral height, and kyphosis, severely limiting the mobility of patients and causing a serious impact on the quality of life of elderly patients [9–11]. Traditional treatment methods such as medication and bracing cannot effectively relieve pain and even further aggravate the degree of osteoporosis. The mortality rate of patients with OVCF treated with conventional treatment methods has been reported to be as high as about 30% [12]. Currently, PVP has become an effective clinical treatment for OVCF, which can achieve rapid pain relief and promote early functional recovery [13]. However, not all patients can achieve satisfactory results after surgery, and some patients still experience poor pain relief and reinforced vertebral re-collapse after treatment. Literature reported that the success rate of PVP surgery for OVCF was 89% to 93%, indicating that there is still room for improvement in the clinical efficacy of such surgery [14]. However, there are many factors that affect the efficacy of PVP surgery. Relevant studies have confirmed that the distribution of bone cement is one of the main factors affecting efficacy [1, 15]. Bone cement injected into the vertebral body will form different cement distributions due to different surrounding pressures. When the distribution of bone cement is limited to one side of the vertebral body, it is easy to cause an uneven local force on the injured vertebrae, which increases the risk of spinal instability and the probability of collapse of the injured vertebrae [16].

At present, there are no uniform evaluation criteria for assessing the distribution of bone cement within the vertebral body. Tanigawa et al. [17] divided the bone cement into "compact mass" types and "sponge dispersion" types according to the morphological characteristics of bone cement and found that the morphology of the bone cement was not associated with improvement in clinical symptoms, but the incidence of new vertebral fractures, especially fractures of adjacent vertebrae, was significantly higher in the "compact mass" type than in the "spongy dispersion" type. Nam et al. [18] divided the distribution of bone cement into three types: unilateral, bilateral inhomogeneous, and bilaterally homogeneous. Zhang et al. [15] classified the distribution of bone cement into four groups in the sagittal position: the group that contacts both the upper and lower endplate; the group that contacts the upper endplate; the group that contacts the lower endplate; and the intermediate group. The above classification methods play a certain role in evaluating the distribution of bone cement after vertebroplasty and predicting the efficacy. However, due to the lack of postoperative CT examination, the above classification methods are greatly affected by subjective factors and cannot truly and objectively reflect cement distribution and morphology in the vertebral body. Therefore, we evaluated the distribution of intravertebral cement from multiple planes based on postoperative x-ray, CT, and 3D reconstruction, and used a scoring system to calculate the distribution range and score of intravertebral cement, which is more objective and accurate than using two-dimensional images, such as x-ray films alone.

In the present study, there were significant differences in BMD, CDV, cement leakage, and VF% between the two groups. Vertebral stiffness and strength were found to be dependent on BMD in an experimental biomechanical study of multilevel segments with polymethylmethacrylate augmentation at levels 0, 1, 2, and 3 [19]. The results of univariate analysis showed that BMD was an influencing factor of bone cement distribution, indicating that BMD had a significant effect on the dispersion of cement in the vertebral body. This may be because BMD reflects the quality and quantity of vertebral bone. The larger BMD indicates the greater number of bone trabeculae per unit area. At the same time, the thicker the bone trabeculae, the more bone mineral content, and the smaller the bone trabecular gap, the less bone cement can be accommodated per unit volume. Injecting the same volume of bone cement requires a wider range of spatial dispersion under the same fluidity state. In contrast, the lower the BMD, the smaller the bone trabeculae, the less bone mineral content, the larger the bone trabecular gap, the more bone cement can be accommodated per unit volume, and thus the smaller the range of bone cement dispersion within it. At the same time, the higher the bone density, the greater the resistance to vertebroplasty, which often requires higher pressure to push the cement into the vertebral body. Liu et al. [20] showed that when the same volume of bone cement was injected into the same vertebral segment, the higher the bone density, the more extensive the distribution of bone cement. The results of multivariate logistic regression analysis showed that good bone cement distribution after PVP was positively correlated with BMD (*p* = 0.022), which indicated that better BMD could improve the dispersion of bone cement within the vertebral body.

The bone cement injection amount is one-sided as an indicator reflecting bone cement dispersion [21]. A large amount of bone cement injected does not necessarily result in wide dispersion. CDV refers to the dispersion of cement within the vertebral body along the bone trabeculae or the gap between the fracture lines, forming a 3D spatial structure composed of cement, bone trabeculae, and their gaps. CDV is a valid indicator to measure the degree of bone cement dispersion [22, 23], which can more reasonably reflect the dispersion of bone cement within the vertebral body. Therefore, in this study, we used the Mimics 3D reconstruction method to calculate CDV with more accuracy and science. Additionally, the results of the univariate analysis showed that cement leakage was a factor influencing cement distribution (*p* < 0.05). This suggests that the amount of cement injected into the vertebral body may be altered when leakage occurs, leading to inadequate cement distribution. However, we found that cement leakage was not an independent factor affecting cement distribution when included in a multivariate logistic regression analysis (*p* > 0.05). We believe that this may be related to the limited number of cases included in our study. At the same time, all cement used in this study was high viscosity bone cement, which may also have a certain effect on preventing cement leakage [24].

In vitro experiments and biomechanical studies have shown that minimal VF% is required to restore the mechanical properties of vertebral compression fractures. Nieuwenhuijse et al. [25] believed that the most clinically significant VF% was 24%, which could achieve a good analgesic effect and avoid bone cement leakage and other related complications. Jin et al. [26] concluded that to ensure surgical efficacy and reduce complications, the VF% should be at least 11.64%. They both demonstrated that using VF% as an evaluation criterion to predict postoperative efficacy is a good indicator. The VF% used in this study was the ratio of CDV to VBV. The results of univariate analysis showed that the VF% in group A was significantly lower than that in group B, and the difference was statistically significant (*p* < 0.001). Meanwhile, the results of multivariate analysis showed that the VF% was an independent influencing factor of bone cement distribution. The concept of VF% not only includes the factors of bone cement injection dose but also fully considers the 3D distribution range of bone cement in the vertebral body and the size of the vertebral body volume. It also involves the vertebral body BMD factor, which can better judge the intraoperative bone cement distribution.

In this study, based on postoperative x-ray, CT, and Mimics 3D reconstruction functions, we divided the vertebral body into different areas from the anteroposterior, lateral, and horizontal positions, respectively, and evaluated the distribution of bone cement in the vertebral body from multiple planes. The distribution of bone cement as well as CDV, VBV, and VF% were accurately observed and calculated. Multivariate logistic regression analysis showed that VF% was an independent influence on the distribution of bone cement after PVP (OR = 1.259, 95% CI 1.135 to 1.396, *p* < 0.001). The bone cement should be distributed as widely and evenly as possible during the operation to obtain a good prognosis. By establishing the ROC curve, we found that an extensive distribution of bone cement could be obtained when the VF% reached 28.58%, with a sensitivity of 72.2% and a specificity of 91.7%. Finally, the correlation analysis showed a strong positive correlation between the cement distribution score and the VF% (*r* = 0.895, *p* < 0.001), indicating the feasibility and validity of the cement distribution score calculated based on postoperative x-ray and CT for assessing the VF%, suggesting that an appropriate increase in the cement distribution score could promote the VF% of OVCF patients after PVP.

In actual clinical work, in patients undergoing PVP for OVCF, in order to obtain an extensive distribution of bone cement, attention should be paid to: (1) The location of the puncture entry point should be accurate; (2) Bilateral puncture should be selected as much as possible; (3) The timing of bone cement injection should be determined by evaluating the BMD of the injured vertebrae by preoperative imaging; (4) The target point of bone cement injection should be located in the center of the fracture as much as possible, rather than the anterior 1/3 of the vertebral body in all cases.

Our study has several limitations. Firstly, the sample size was small, the follow-up time was short, and there was a lack of multi-center prospective randomized controlled studies. Secondly, we only studied the minimum value of the extensive distribution of bone cement, but the leakage of bone cement may increase with an increase in VF%. Furthermore, we did not consider the contact between bone cement and the endplate in this study, which may be a weakness in our research methodology and could potentially have an impact on our study results. This will need to be further validated in future research. Finally, we found that as the amount of cement filling increases, the distribution score also increases, and the probability of cement leakage may also increase. Therefore, a higher distribution score does not necessarily indicate a better outcome. However, we did not consider the maximum distribution score when leakage occurred in our study. In future studies, we will further expand the sample size, conduct a prospective and multi-center study, and determine the maximum VF% and distribution score when cement leakage occurs.

## Conclusion

The vertebral BMD is closely related to the extent of bone cement distribution in the vertebral body, and the higher the BMD, the greater the extent of bone cement distribution. Meanwhile, both BMD and VF% are important independent influencing factors of bone cement distribution. Extensive bone cement distribution can be achieved when the VF% reaches 28.58%.

## Data Availability

The datasets used and/or analyzed during the current study are available from the corresponding author on reasonable request.

## Abbreviations

OVCF: Osteoporotic vertebral compression fracture
PVP: Percutaneous vertebroplasty
PKP: Percutaneous kyphoplasty
BMI: Body mass index
BMD: Bone mineral density
CDV: Cement dispersion volume
VBV: Vertebral body volume
VF%: Volume fraction percentage
ROC: Receiver operating characteristic
AUC: Area under the curve
MRI: Magnetic resonance imaging
CI: Confidence interval
OR: Odds ratio

## References

[1] Lin J, Qian L, Jiang C, Chen X, Feng F, Lao L (2018). Bone cement distribution is a potential predictor to the reconstructive effects of unilateral percutaneous kyphoplasty in OVCFs: a retrospective study. J Orthop Surg Res.

[2] Wardlaw D, Cummings SR, Van Meirhaeghe J, Bastian L, Tillman JB, Ranstam J, Eastell R, Shabe P, Talmadge K, Boonen S (2009). Efficacy and safety of balloon kyphoplasty compared with non-surgical care for vertebral compression fracture (FREE): a randomised controlled trial. Lancet.

[3] Kendler DL, Bauer DC, Davison KS, Dian L, Hanley DA, Harris ST, McClung MR, Miller PD, Schousboe JT, Yuen CK, Lewiecki EM (2016). Vertebral fractures: clinical importance and management. Am J Med.

[4] Ballane G, Cauley JA, Luckey MM, El-Hajj FG (2017). Worldwide prevalence and incidence of osteoporotic vertebral fractures. Osteoporos Int.

[5] Wang B, Guo H, Yuan L, Huang D, Zhang H, Hao D (2016). A prospective randomized controlled study comparing the pain relief in patients with osteoporotic vertebral compression fractures with the use of vertebroplasty or facet blocking. Eur Spine J.

[6] Yang EZ, Xu JG, Huang GZ, Xiao WZ, Liu XK, Zeng BF, Lian XF (2016). Percutaneous vertebroplasty versus conservative treatment in aged patients with acute osteoporotic vertebral compression fractures: a prospective randomized controlled clinical study. Spine (Phila Pa 1976).

[7] Ding X, Zhang Q, Zhao Y, Wang J (2022). Location and effect of bone cement in percutaneous vertebroplasty for osteoporotic vertebral compression fractures. Biomed Res Int.

[8] Liu C, Song W, Liu C, Liang K, Zhang K, Li Y (2019). EvaIuation and influencing factor anaIysis of bone cement distribution after percutaneous vertebroplasty. Chin J Spine SpinaI Cord.

[9] Chen D, An ZQ, Song S, Tang JF, Qin H (2014). Percutaneous vertebroplasty compared with conservative treatment in patients with chronic painful osteoporotic spinal fractures. J Clin Neurosci.

[10] Gao X, Du J, Gao L, Hao D, Hui H, He B, Yan L (2022). Risk factors for bone cement displacement after percutaneous vertebral augmentation for osteoporotic vertebral compression fractures. Front Surg.

[11] Zhang ZL, Yang JS, Hao DJ, Liu TJ, Jing QM (2021). Risk factors for new vertebral fracture after percutaneous vertebroplasty for osteoporotic vertebral compression fractures. Clin Interv Aging.

[12] Lee YK, Jang S, Jang S, Lee HJ, Park C, Ha YC, Kim DY (2012). Mortality after vertebral fracture in Korea: analysis of the National Claim Registry. Osteoporos Int.

[13] Stadelmann VA, Zderic I, Baur A, Unholz C, Eberli U, Gueorguiev B (2016). Composite time-lapse computed tomography and micro finite element simulations: A new imaging approach for characterizing cement flows and mechanical benefits of vertebroplasty. Med Eng Phys.

[14] McConnell CT, Wippold FJ, Ray CE, Weissman BN, Angevine PD, Fries IB, Holly LT, Kapoor BS, Lorenz JM, Luchs JS, O'Toole JE, Patel ND, Roth CJ, Rubin DA (2014). ACR appropriateness criteria management of vertebral compression fractures. J Am Coll Radiol.

[15] Zhang L, Wang Q, Wang L, Shen J, Zhang Q, Sun C (2017). Bone cement distribution in the vertebral body affects chances of recompression after percutaneous vertebroplasty treatment in elderly patients with osteoporotic vertebral compression fractures. Clin Interv Aging.

[16] Zhou C, Liao Y, Huang S, Li H, Zhu Z, Zheng L, Wang B, Wang Y (2022). Effect of cement distribution type on clinical outcome after percutaneous vertebroplasty for osteoporotic vertebral compression fractures in the aging population. Front Surg.

[17] Tanigawa N, Komemushi A, Kariya S, Kojima H, Shomura Y, Omura N, Sawada S (2007). Relationship between cement distribution pattern and new compression fracture after percutaneous vertebroplasty. AJR Am J Roentgenol.

[18] Nam HG, Jeong JH, Shin IY, Moon SM, Hwang HS (2012). Clinical effects and radiological results of vertebroplasty: over a 2-year follow-up period. Korean J Spine.

[19] Kayanja MM, Schlenk R, Togawa D, Ferrara L, Lieberman I (2006). The biomechanics of 1, 2, and 3 levels of vertebral augmentation with polymethylmethacrylate in multilevel spinal segments. Spine (Phila Pa 1976).

[20] Liu J, Liu Z, Luo J, Gong L, Cui Y, Song Q, Xiao PF, Zhou Y (2019). Influence of vertebral bone mineral density on total dispersion volume of bone cement in vertebroplasty. Medicine (Baltimore).

[21] Mousavi P, Roth S, Finkelstein J, Cheung G, Whyne C (2003). Volumetric quantification of cement leakage following percutaneous vertebroplasty in metastatic and osteoporotic vertebrae. J Neurosurg.

[22] Li H, Yang DL, Ma L, Wang H, Ding WY, Yang SD (2017). Risk factors associated with adjacent vertebral compression fracture following percutaneous vertebroplasty after menopause: a retrospective study. Med Sci Monit.

[23] Ye LQ, Liang D, Jiang XB, Yao ZS, Lu H, Qiu T, Yu WB, Mo L, Zhang SC, Jin DX (2018). Risk factors for the occurrence of insufficient cement distribution in the fractured area after percutaneous vertebroplasty in osteoporotic vertebral compression fractures. Pain Physician.

[24] Wang Q, Sun C, Zhang L, Wang L, Ji Q, Min N, Yin Z (2022). High- versus low-viscosity cement vertebroplasty and kyphoplasty for osteoporotic vertebral compression fracture: a meta-analysis. Eur Spine J.

[25] Nieuwenhuijse MJ, Bollen L, van Erkel AR, Dijkstra PD (2012). Optimal intravertebral cement volume in percutaneous vertebroplasty for painful osteoporotic vertebral compression fractures. Spine (Phila Pa 1976).

[26] Jin YJ, Yoon SH, Park KW, Chung SK, Kim KJ, Yeom JS, Kim HJ (2011). The volumetric analysis of cement in vertebroplasty: relationship with clinical outcome and complications. Spine (Phila Pa 1976).

